# What Lies Behind Substantial Differences in COVID-19 Vaccination Rates Between EU Member States?

**DOI:** 10.3389/fpubh.2022.858265

**Published:** 2022-05-26

**Authors:** Josip Franic

**Affiliations:** Institute of Public Finance, Zagreb, Croatia

**Keywords:** COVID-19, vaccine hesitancy, anti-vaccinationism, socialist legacy, EU, multilevel modeling

## Abstract

**Background:**

Despite the billions of doses at disposal, less than three-quarters of EU citizens received a COVID-19 vaccine by the end of 2021. The situation is particularly worrying in transition societies, which experience much stronger opposition to vaccination compared to their Western counterparts. To understand whether and to what extent this has to do with the socialist legacy, in this paper we explore wider economic, political, and cultural determinants of the COVID-19 vaccine uptake in the EU.

**Methods:**

Data from Flash Eurobarometer 494 conducted in May 2021 were used to model the attitudes of EU citizens toward COVID-19 vaccination. Based on their views and intentions, each of 26,106 survey participants was allocated into one of the following categories: (1) already vaccinated/plan to get vaccinated; (2) indecisive; (3) refuse vaccination. Multilevel multinomial logit was employed to understand what underlies the reasoning of each group.

**Results:**

The survey revealed that 13.4% of Europeans planned to delay vaccination against COVID-19, while 11.2% did not intend to get vaccinated. Although numerous demographic and socio-economic factors jointly shape their viewpoints, it is trust (in the authorities, science, peers, and online social networks above all) that strongly dominates citizens' reasoning. Given that most transition societies are witnessing the pandemic of distrust at various levels, this seemingly unrelated feature appears to be vital in explaining why newer member states record lower vaccination rates. Education was also found to play a pivotal role, which is reflected in an individual's ability to critically assess information from various sources.

**Conclusion:**

The study results clearly illustrate how long-lasting structural problems (specific for, but not confined to, transition countries) can manifest themselves in unforeseen circumstances if left unaddressed. It is hence of vital importance to learn the lesson and prevent similar issues in the future. Above all, this would require wide-ranging reforms aiming to repair the imperceptible psychological contract between citizens and the state authorities.

## Introduction

Notwithstanding the rising general aversion to vaccination^1^, the approval of the Comirnaty vaccine on December 21, 2020, was celebrated as a turning point in the fight against Coronavirus disease 2019 (COVID-19) in the European Union (EU) (2, 3). Scientists, medical experts, politicians, and the wider community mistakenly assumed that common sense in combination with economic, social, and psychological distress caused by the pandemic would take the victory over fear, skepticism, and conspiracy theories (4–6). On the contrary, the term “herd immunity,” which dominated media reports and political speeches at that time, has gradually evaporated from the public sphere during the following year. With less than three-quarters of EU citizens receiving their dose(s) by the end of 2021 (7), the virus managed to survive within the population and eventually mutate to a worrying degree. As a result, 2 years after the onset of the pandemic the member states have witnessed record numbers of new infections on a day-to-day basis owing to the fast-spreading Omicron variant.

**Figure 1:**
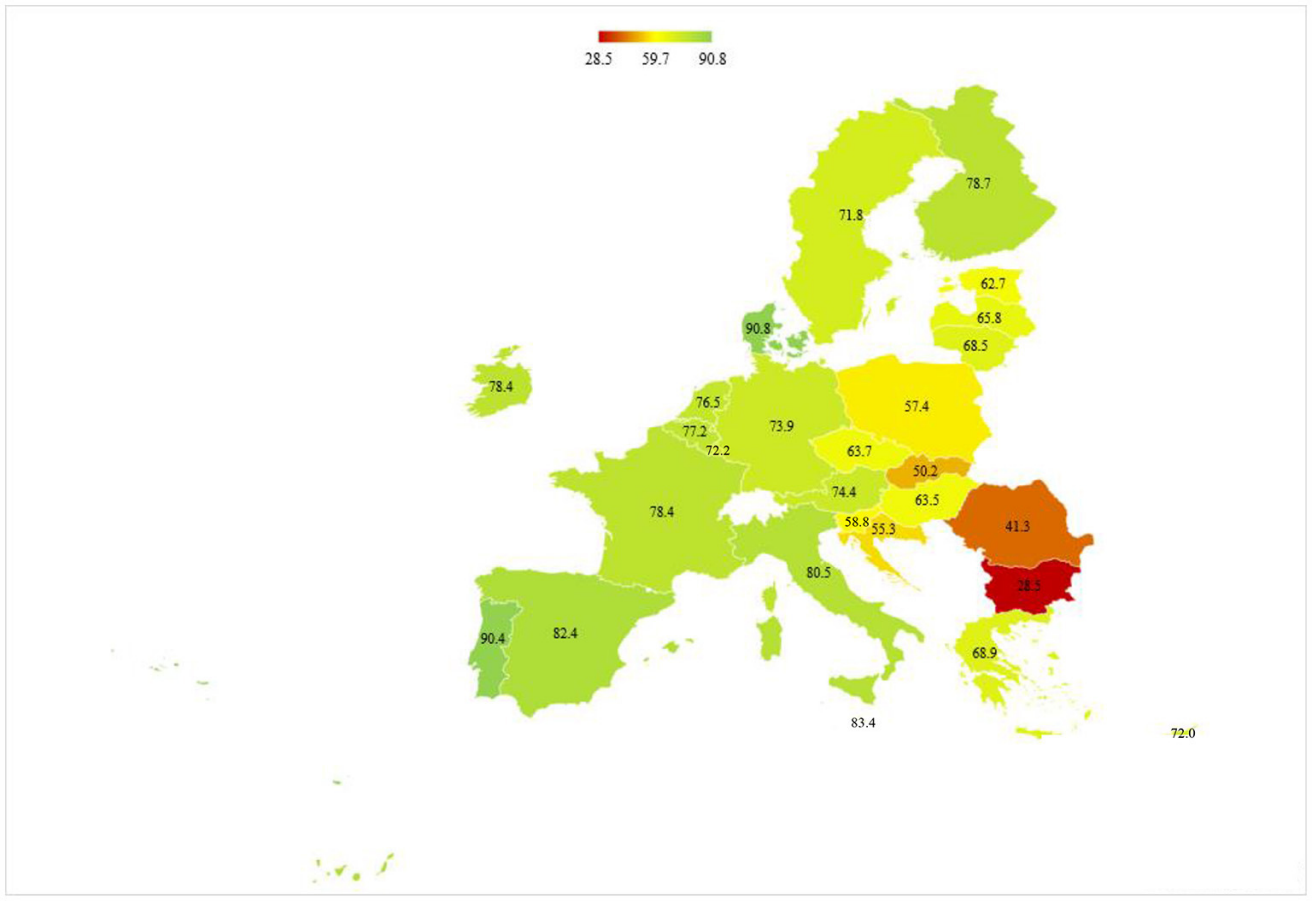
Vaccination rates across the EU, % of the population. The figure shows the percentages of the total population receiving at least one dose of the COVID-19 vaccine by January 4, 2022. Source: Author's own calculations based on data from ECDC (7).

However, a closer look at the official data by the European Center for Disease Prevention and Control (ECDC) reveals noticeable discrepancies between EU countries concerning vaccination rates. The share of the population receiving at least one dose of vaccine against COVID-19 ranges from as low as 28.5% in Bulgaria to as high as 90.8% in Denmark (Figure 1). In fact, post-socialist countries lag far behind in this regard. For instance, while the majority of citizens in Portugal, Malta, and Spain have been immunized to date, the fight against the disease in Romania, Slovak Republic, and Croatia has been impeded by disturbingly low coverage rates (accounting for 41.3%, 50.2%, and 55.3%, respectively). Given noteworthy coordination in the acquisition and distribution of vaccines at the EU level on the one hand, and ease of access for all EU citizens on the other, the difference in vaccine acceptance appears to be the only reasonable explanation for this state of affairs.

Whether and how the attitudes toward vaccination against COVID-19 are exactly related to the socialist legacy, however, has not been evaluated so far. Previous studies on the matter were mainly concerned with socio-demographic aspects of vaccine uptake in the EU and safety concerns related to the speed of vaccine development (8, 9), while cultural, political, and economic determinants were left aside. It is precisely this gap we aim to fill in the rest of this paper. Specifically, the idea is to evaluate to what extent the standpoints and actions of EU citizens are shaped by personal characteristics and to what extent they arose from the environment in which an individual lives and operates.

To do so, we build upon the results of existing studies from around the world, which have identified a range of factors underlying views, opinions, and intentions of citizens regarding COVID-19 vaccination. In addition to demographic peculiarities, such as gender (10–12), age (8, 13, 14), and ethnicity (15, 16), it was found that one's formal and informal education also play important roles in this respect. More precisely, evidence suggests that people with a university diploma generally demonstrate lower vaccine hesitancy than low-skilled individuals (14, 17, 18). Moreover, resistance to vaccination appears to have a lot to do with reliance on unverified sources of information (e.g. online social networks), and in particular with susceptibility to conspiracy theories (14, 19, 20). Political orientation and religion are also significant determinants, as anti-vaccination sentiment was found to be more ingrained among conservative voters and highly religious people (15, 21, 22).

Some recent inquiries as well revealed that individuals who have experienced severe psychological, economic, and/or health distress during the pandemic are more open to vaccination, and the same applies to those expressing pro-social behavior (9, 23, 24). Finally, and most importantly, a number of studies identified trust as the key piece of this compound puzzle. This applies not only to the assessment of the tools chosen by the authorities to combat the ongoing pandemic (9, 20, 22), but also to a general confidence in the ruling elites, modern science, the media, and fellow citizens (8, 9, 25). Given that a growing body of research has identified the “pandemic of distrust” as the main factor explaining the rise of “anti-systemic behavior” in transition societies^2^, this issue owes to be given due attention in our case as well.

To sum up, in line with the findings from previous studies on the matter, the following five hypotheses will be evaluated in the rest of this paper:

Hypothesis 1**:** The readiness of EU citizens to receive a COVID-19 vaccine is closely related to the effect the pandemic has had on their well-being.
Hypothesis 2: Substantial differences in vaccine-acceptance rates can be ascribed to the uneven quality of both formal and informal education across the EU.
Hypothesis 3: Strong opposition to COVID-19 vaccination in some EU countries reflects the low quality of the psychological contract between citizens and the authorities.
Hypothesis 4: Individual's attitude toward vaccination is substantially shaped by the strength of social ties within their community.
Hypothesis 5: Individual's (un)willingness to get vaccinated is under a strong influence of their political and religious views.

The ultimate goal of this research article goes beyond informing and assisting the current vaccination campaigns, as the intention is to shed light on certain leftovers from previous political and economic regimes whose adverse effects could easily surpass the current anti-vaccination movement if not properly addressed. In addition to advancing our knowledge of the mechanisms underlying vaccination attitudes, the study is, therefore, also expected to resonate in other academic fields. This particularly applies to research on the issues of trust, governance, social cohesion, and quality of education, which are often neglected in discussions of the challenges modern healthcare systems (and societies in general) are facing. When it comes to methodological advancements, to the best of the author's knowledge this paper represents the very first attempt to explore non-medical factors responsible for such extensive discrepancies in vaccination rates at the EU level.

To achieve the enumerated objectives, the next section describes the data used and statistical methods applied to test research hypotheses, while Section 3 brings the results of the conducted analysis. This is followed by a discussion and concluding remarks, which are given in the last section of the paper.

## Methods

The analysis is grounded on data from Flash Eurobarometer 494: “Attitudes on vaccination against COVID-19.” This survey, conducted in May 2021 on a sample of 26,106 individuals, represents the first and thus far the only publicly available EU-wide inquiry into the matter. Approximately 1,000 respondents above the age of 15 were recruited following the quota sampling approach in the majority of member states. The exceptions were Malta, Cyprus, and Luxembourg, with the final samples accounting for 515, 513, and 511 respectively^3^.

Among a range of questions related to the pandemic, each interviewee was asked when they would like to get vaccinated against COVID-19, with the following options offered: (1) as soon as possible; (2) sometime in 2021; (3) later; (4) never; (5) already vaccinated; (6) do not know; (7) prefer not to answer^4^. Since the survey took place at the moment when vaccines were not fully accessible (i.e. in most countries the focus was still on the elderly and individuals with comorbidities), it is rational to assume that interviewees stating “as soon as possible” or “sometime in 2021” had received their dose(s) by the end of 2021. For the purpose of the analysis, we hence made no distinction between individuals from categories (1), (2), and (5) above. Following the approach applied in similar studies (10, 18), besides this “pro-vaccination” group we also distinguish indecisive individuals (answers “later” and “do not know”) and those who refuse vaccination (answer “never”). On the other hand, the option “prefer not to answer” was treated as a missing response and accordingly imputed using the Markov Chain Monte Carlo method^5^.

The preliminary tests showed that proportional odds assumption does not hold, which implies that individuals not intending to get vaccinated significantly differ from the indecisive ones in terms of the mechanisms underlying their reasoning. Following this, multinomial logistic regression appeared as a natural choice in the search for the factors explaining variability in vaccination rates across the EU. The results of the null model revealed that 9.3% (Wald test = 3.282, *P* < 0.001) of variance in likelihood to delay vaccination and 15.1% (Wald test = 3.203, *P* < 0 .001) of variance in likelihood to refuse vaccination can be ascribed to the particularities of the country in which a respondent lives. This highlighted the need to pursue a multilevel approach so as to obtain unbiased results. Given this, we made use of the two-level random intercept multinomial logit model, which is defined as follows:


$$\begin{array}{lll} \ln(\frac{P(y_{i}=m)}{P(y_{i}=0)}) & = & \beta_{0j}+\overset{k=1}{\underset{K}{\sum^{\text{​}}}}\beta_{k}X_{ijk}+\overset{s=1}{\underset{S}{\sum^{\text{​}}}}\gamma_{s}Z_{js\;},m=1,2\\ \;\beta_{0j} & = & \beta_{0}+u_{j},\;\;\;j=1,2,\ldots ,\;27 \end{array}$$

where *y*_*i*_ represents the value of the dependent variable for an individual *i* (0: Already vaccinated/Plan to get vaccinated; 1: Indecisive; 2: Refuse vaccination) and *X*_1_-*X*_*K*_ are individual-level covariates exerting effects β_1_-β_*K*_ on the dependent variable. Since data are given on two levels, the intercept value is allowed to vary from country to country by including the group-level residuals *u*_j_ ~ *N*(0, σ^2^).

Besides controlling for the hierarchical nature of data, the multilevel modeling also offered the opportunity to explore which country-level factors (*Z*_1_−*Z*_*S*_ in the equation above) are responsible for the aforementioned variability in vaccination coverage. To exploit the full potential of this research paradigm, a mix of individual-level (i.e. level-1) variables available directly from the survey and country-level (level-2) variables compiled from other sources were hence used in the analysis.

Specifically, to evaluate Hypothesis 1, the following explanatory variables were included: binary indicator designating whether a respondent had been seriously ill because of COVID-19, binary indicator capturing one's subjective perception about the ability to avoid being infected by COVID-19 without vaccination (level-1 variables), the number of cumulative COVID-19 deaths in a country (adjusted for population size), the measure of the stringency of national policies to suppress COVID-19 (on a scale from 0 to 100), and GPD growth rates for 2020 (level-2 variables)^6^. On the other hand, age when finishing education (level-1) and average PISA scores for 2018 (a proxy for the overall quality of the national education system, level-2 variable) were used to test whether formal education is important in this respect (Hypothesis 2). To further explore how the individual's ability to critically assess information influences their viewpoints, we also included level-1 binary variables signifying whether they find online social networks and media as trustworthy. These two were supplemented with a level-2 variable denoting the portion of the population that tends to trust conspiracy theories.

Turning to the role of the psychological contract between citizens and the authorities (Hypothesis 3), included are also binary variables indicating whether a person thinks public authorities have been sufficiently transparent about COVID-19 vaccines and whether they are satisfied with the way the government has handled the vaccination strategy (level-1). The broader effects of trust were scrutinized through interval variables measuring the overall support for the work of the government (not specific to COVID-19), the share of the population contented with the way democracy works in their country, and the share of citizens who distrust science (level-2).

To check how views and experiences of people in their surroundings affect one's standpoints (Hypothesis 4), the following level-1 covariates were also examined: binary variable indicating whether an individual relies on their colleagues, friends, and family when seeking information on COVID-19 vaccines, binary variable denoting whether they have people from close social circle who have been seriously ill because of COVID-19 and the categorical variable for the total number of adults in the household. To explore the role of social cohesion on a wider scale, we also included a level-2 variable representing the percentage of citizens who feel very attached to their country.

The effects of religion and political orientation (Hypothesis 5) were examined using country-level variables that indicate the percentage of people attending religious services at least once a week and the average positioning of the population on the political scale (where 1 is fully left and 10 is fully right). Finally, to control for other factors known to affect COVID-19 vaccine uptake, we also included age, gender, place of residence, migrant status, and history of previous vaccinations as explanatory variables in the models.

To sum up, a total of 12 models were constructed in a cumulative model-building fashion (25, 26). Model 1 comprises individual-level covariates only, while Models 2–12 sequentially include each of the enumerated country-level variables. The following section brings the most important findings from the conducted analysis.

## Results

**Figure 2:**
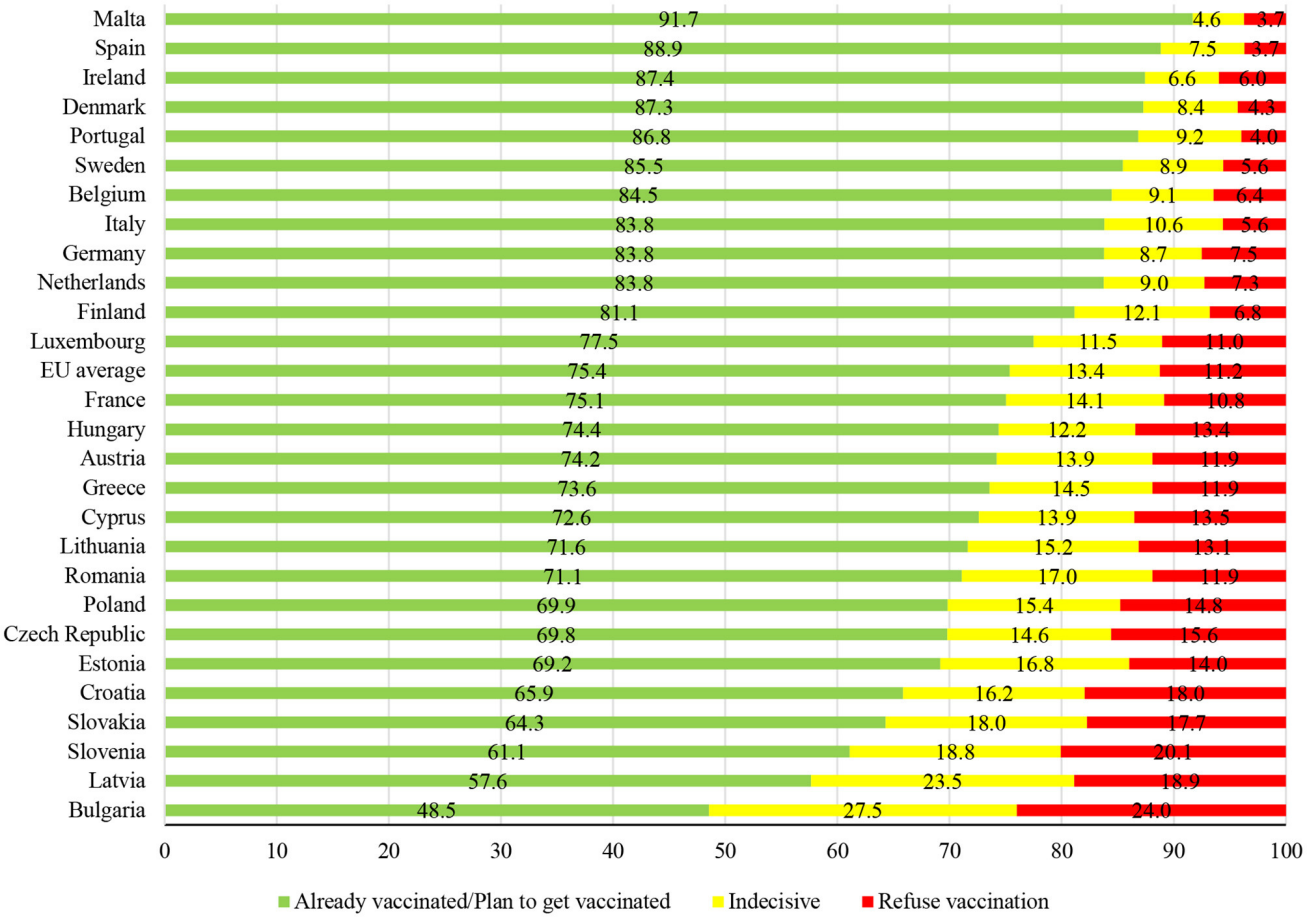
Attitudes toward vaccination against COVID-19 across the EU, % of population. Source: Author's own calculations based on data from Flash Eurobarometer 494.

As expected, the survey revealed that people from transition societies indeed exhibit much lower enthusiasm for vaccination than their western counterparts (Figure 2). More importantly, the ordering of countries based on the share of the population willing to get vaccinated in a due time closely matches the one based on true vaccination rates (as illustrated in Figure 1). For instance, survey respondents from Malta, Spain, Denmark, and Portugal were highly supportive of this strategy to combat the virus, which translated into high coverage rates at the end of 2021. The situation is diametrically opposite in post-socialist countries, where a substantial portion of residents either fully oppose vaccination or are indecisive.

To understand why this is so, Table 1 presents the results of the multilevel multinomial logit model. Starting with Hypothesis 1, our findings challenge the hypothesized link between pandemic fatigue and the readiness of EU citizens to receive COVID-19 vaccines. Specifically, cumulative death rates, GDP growth rates, and the stringency of restrictions appear not to be particularly helpful in explaining why certain member states were more successful in vaccination. This, however, does not mean that Hypothesis 1 should be rejected. Quite the opposite, the findings on the remaining two variables highlight egocentrism as the key factor in this respect. More precisely, persons confident in their ability to avoid infection were found to be less keen on vaccination. The same is true for the ones who recently recovered from COVID-19. While expected for individuals wishing to delay vaccination (owing to a natural immunity gained), in the case of those opposing vaccination the latter actually points to important knowledge gaps.

**Table 1:** Results of the multilevel multinomial logit.

	**Model 1**		**Model 2**		**Model 3**		**Model 4**	
	**Indecisive**	**Refuse vaccination**	**Indecisive**	**Refuse vaccination**	**Indecisive**	**Refuse vaccination**	**Indecisive**	**Refuse vaccination**
**Intercept**	1.542^***^ (0.161)	−1.291^***^ (0.189)	1.531^***^ (0.162)	−1.276^***^ (0.188)	1.536^***^ (0.159)	−1.295^***^ (0.188)	1.545^***^ (0.161)	−1.295^***^ (0.188)
**Gender (RC: Male)**	0.256^***^ (0.038)	0.186^***^ (0.042)	0.257^***^ (0.038)	0.187^***^ (0.042)	0.256^***^ (0.038)	0.186^***^ (0.042)	0.256^***^ (0.038)	0.186^***^ (0.042)
**Age (group centered)**	−0.022^***^ (0.001)	−0.015^***^ (0.001)	−0.022^***^ (0.001)	−0.015^***^ (0.001)	−0.022^***^ (0.001)	−0.015^***^ (0.001)	−0.022^***^ (0.001)	−0.015^***^ (0.001)
**Age when finalizing education (RC:** ** <16)**								
16–19	−0.099 (0.112)	−0.195 (0.118)	−0.101 (0.112)	−0.201 (0.118)	−0.104 (0.112)	−0.193 (0.118)	−0.099 (0.112)	−0.197 (0.118)
20+	−0.302^**^ (0.112)	−0.445^***^ (0.118)	−0.307^**^ (0.112)	−0.453^***^ (0.118)	−0.306^**^ (0.112)	−0.446^***^ (0.118)	−0.300^**^ (0.112)	−0.448^***^ (0.118)
Still studying	−0.417^***^ (0.122)	−0.676^***^ (0.131)	−0.421^***^ (0.122)	−0.685^***^ (0.131)	−0.420^***^ (0.122)	−0.678^***^ (0.131)	−0.416^***^ (0.122)	−0.678^***^ (0.132)
Never had formal education	−0.005 (0.153)	−0.126 (0.169)	−0.011 (0.153)	−0.129 (0.169)	−0.008 (0.153)	−0.125 (0.169)	−0.001 (0.154)	−0.128 (0.169)
**Number of adults in the household (group centered)**	−0.006 (0.015)	0.032^*^ (0.016)	−0.006 (0.015)	0.032^*^ (0.016)	−0.006 (0.015)	0.033^*^ (0.016)	−0.006 (0.015)	0.033^*^ (0.016)
**Place of residence (RC: Rural area)**								
Small or middle-sized town	0.022 (0.048)	−0.252^***^ (0.051)	0.021 (0.048)	−0.255^***^ (0.052)	0.021 (0.048)	−0.254^***^ (0.052)	0.022 (0.048)	−0.253^***^ (0.051)
Large town	−0.165^**^ (0.051)	−0.415^***^ (0.055)	−0.168^**^ (0.051)	−0.418^***^ (0.055)	−0.165^**^ (0.051)	−0.417^***^ (0.055)	−0.165^**^ (0.051)	−0.415^***^ (0.055)
**Living abroad**	0.360^***^ (0.100)	0.180 (0.121)	0.357^***^ (0.100)	0.186 (0.121)	0.355^***^ (0.100)	0.182 (0.121)	0.360^***^ (0.100)	0.179 (0.121)
**Vaccinated in adult age**	−0.510^***^ (0.040)	−0.717^***^ (0.044)	−0.514^***^ (0.040)	−0.721^***^ (0.044)	−0.509^***^ (0.040)	−0.720^***^ (0.044)	−0.510^***^ (0.040)	−0.717^***^ (0.044)
**Seriously ill because of COVID-19**	0.277 ^***^ (0.054)	0.225^***^ (0.062)	0.278^***^ (0.055)	0.223^***^ (0.062)	0.276 ^***^ (0.054)	0.226^***^ (0.062)	0.277 ^***^ (0.054)	0.224^***^ (0.062)
**Knowing people who were seriously ill because of COVID-19**	−0.411^***^ (0.045)	−0.698^***^ (0.048)	−0.412^***^ (0.046)	−0.705^***^ (0.048)	−0.412^***^ (0.045)	−0.701^***^ (0.048)	−0.410^***^ (0.045)	−0.699^***^ (0.048)
**Satisfaction with the way government has handled the vaccination strategy**	−0.745^***^ (0.040)	−1.497^***^ (0.050)	−0.749^***^ (0.040)	−1.502^***^ (0.050)	−0.746^***^ (0.040)	−1.501^***^ (0.050)	−0.745^***^ (0.040)	−1.499^***^ (0.050)
**Public authorities not sufficiently transparent about COVID-19 vaccines**	0.349^***^ (0.044)	0.413^***^ (0.051)	0.350^***^ (0.044)	0.414^***^ (0.051)	0.349^***^ (0.044)	0.414^***^ (0.051)	0.348^***^ (0.044)	0.414^***^ (0.051)
**Websites provide reliable information on COVID-19 vaccines**	0.029 (0.068)	0.194^**^ (0.072)	0.028 (0.068)	0.195^**^ (0.072)	0.029 (0.068)	0.194^**^ (0.072)	0.029 (0.068)	0.193^**^ (0.072)
**Online social networks provide reliable information on COVID-19 vaccines**	0.052 (0.077)	0.391^***^ (0.078)	0.053 (0.077)	0.394^***^ (0.079)	0.054 (0.077)	0.393^***^ (0.077)	0.053 (0.077)	0.392^***^ (0.079)
**Colleagues, friends and family provide reliable information on COVID-19 vaccines**	0.127^**^ (0.048)	−0.249^***^ (0.056)	0.128^**^ (0.048)	−0.249^***^ (0.056)	0.126^**^ (0.048)	−0.250^***^ (0.056)	0.127^**^ (0.048)	−0.251^***^ (0.056)
**Can avoid COVID-19 infection without being vaccinated**	0.969^***^ (0.042)	1.545^***^ (0.052)	0.971^***^ (0.042)	1.551^***^ (0.052)	0.968^***^ (0.042)	1.549^***^ (0.052)	0.969^***^ (0.042)	1.546^***^ (0.052)
**Country-level variables**								
**Cumulative COVID-19 deaths per 100 million people**			0.017 (0.014)	0.028 (0.019)				
**Stringency of national measures to suppress COVID-19**					−0.017 (0.009)	−0.018 (0.013)		
**GDP growth rate for 2020**							0.049 (0.039)	0.075 (0.051)
**Quality of education system**								
**General trust in government**								
**Satisfaction with democracy**								
**Distrust in science**								
**Proneness to conspiracy theories**								
**Political orientation**								
**Religiosity**								
**Social cohesion**								
**σ^2^**	0.256^***^ (0.073)	0.456^***^ (0.128)	0.256^***^ (0.073)	0.441^***^ (0.128)	0.233^***^ (0.067)	0.439^***^ (0.124)	0.249^***^ (0.071)	0.443^***^ (0.125)
**Variance partition coefficient (VPC)**	0.0722	0.1217	0.0722	0.1181	0.0661	0.1177	0.0704	0.1187
**Covariance**	0.333^***^ (0.094)		0.328^***^ (0.092)		0.312^***^ (0.088)		0.323^***^ (0.091)	
	**Model 5**		**Model 6**		**Model 7**		**Model 8**	
	**Indecisive**	**Refuse vaccination**	**Indecisive**	**Refuse vaccination**	**Indecisive**	**Refuse vaccination**	**Indecisive**	**Refuse vaccination**
**Intercept**	1.537^***^ (0.158)	−1.297^***^ (0.187)	1.521^***^ (0.156)	−1.269^***^ (0.181)	1.534^***^ (0.156)	−1.287^***^ (0.181)	1.544^***^ (0.157)	−1.295^***^ (0.185)
**Gender (RC: Male)**	0.256^***^ (0.038)	0.187^***^ (0.042)	0.256^***^ (0.038)	0.186^***^ (0.042)	0.255^***^ (0.038)	0.186^***^ (0.042)	0.255^***^ (0.038)	0.186^***^ (0.042)
**Age (group centered)**	−0.022^***^ (0.001)	−0.015^***^ (0.001)	−0.022^***^ (0.001)	−0.015^***^ (0.001)	−0.022^***^ (0.001)	−0.015^***^ (0.001)	−0.022^***^ (0.001)	−0.015^***^ (0.001)
**Age when finalizing education (RC:** ** <16)**								
16–19	−0.102 (0.112)	−0.199 (0.118)	−0.100 (0.112)	−0.197 (0.118)	−0.098 (0.112)	−0.195 (0.118)	−0.095 (0.112)	−0.195 (0.118)
20+	−0.307^**^ (0.112)	−0.453^***^ (0.118)	−0.303^**^ (0.112)	−0.450^***^ (0.118)	−0.300^**^ (0.112)	−0.445^***^ (0.118)	−0.298^**^ (0.112)	−0.445^***^ (0.118)
Still studying	−0.421^***^ (0.121)	−0.684^***^ (0.131)	−0.418^***^ (0.121)	−0.681^***^ (0.131)	−0.415^***^ (0.121)	−0.677^***^ (0.131)	−0.413^***^ (0.122)	−0.677^***^ (0.131)
Never had formal education	−0.009 (0.153)	−0.133 (0.169)	−0.006 (0.153)	−0.128 (0.169)	−0.003 (0.153)	−0.125 (0.169)	−0.003 (0.153)	−0.127 (0.169)
**Number of adults in the household (group centered)**	−0.007 (0.015)	0.032^*^ (0.016)	−0.006 (0.015)	0.032^*^ (0.016)	−0.007 (0.015)	0.032^*^ (0.016)	−0.007 (0.015)	0.033^*^ (0.016)
**Place of residence (RC: Rural area)**								
Small or middle-sized town	0.019 (0.048)	−0.254^***^ (0.052)	0.021(0.048)	−0.254^***^ (0.051)	0.021 (0.048)	−0.253^***^ (0.051)	0.022 (0.048)	−0.253^***^ (0.051)
Large town	−0.170^**^ (0.051)	−0.418^***^ (0.055)	−0.166^**^ (0.051)	−0.417^***^ (0.055)	−0.165^**^ (0.051)	−0.416^***^ (0.055)	−0.165^**^ (0.051)	−0.416^***^ (0.055)
**Living abroad**	0.356^***^ (0.100)	0.179 (0.121)	0.363^***^ (0.101)	0.189 (0.121)	0.363^***^ (0.101)	0.185 (0.121)	0.361^***^ (0.100)	0.179 (0.121)
**Vaccinated in adult age**	−0.511^***^ (0.040)	−0.723^***^ (0.044)	−0.510^***^ (0.040)	−0.719^***^ (0.044)	−0.508^***^ (0.040)	−0.717^***^ (0.044)	−0.510^***^ (0.040)	−0.718^***^ (0.044)
**Seriously ill because of COVID-19**	0.276 ^***^(0.054)	0.224^***^ (0.062)	0.276 ^***^(0.054)	0.223^***^ (0.062)	0.276 ^***^(0.054)	0.225^***^ (0.062)	0.277 ^***^(0.054)	0.224^***^ (0.062)
**Knowing people who were seriously ill because of COVID-19**	−0.415^***^ (0.045)	−0.704^***^ (0.048)	−0.412^***^ (0.045)	−0.702^***^ (0.048)	−0.411^***^ (0.045)	−0.700^***^ (0.048)	−0.411^***^ (0.045)	−0.699^***^ (0.048)
**Satisfaction with the way government has handled the vaccination strategy**	−0.750^***^ (0.040)	−1.505^***^ (0.051)	−0.744^***^ (0.040)	−1.499^***^ (0.050)	−0.744^***^ (0.040)	−1.498^***^ (0.050)	−0.744^***^ (0.040)	−1.497^***^ (0.050)
**Public authorities not sufficiently transparent about COVID-19 vaccines**	0.347^***^ (0.044)	0.416^***^ (0.051)	0.348^***^ (0.044)	0.413^***^ (0.051)	0.346^***^ (0.044)	0.412^***^ (0.051)	0.347^***^ (0.044)	0.413^***^ (0.051)
**Websites provide reliable information on COVID-19 vaccines**	0.029 (0.068)	0.194^**^ (0.072)	0.030 (0.068)	0.195^**^ (0.072)	0.030 (0.068)	0.194^**^ (0.072)	0.030 (0.068)	0.193^**^ (0.072)
**Online social networks provide reliable information on COVID-19 vaccines**	0.053 (0.077)	0.395^***^ (0.079)	0.053 (0.077)	0.393^***^ (0.078)	0.053 (0.077)	0.392^***^ (0.078)	0.052 (0.077)	0.391^***^ (0.079)
**Colleagues, friends and family provide reliable information on COVID-19 vaccines**	0.128^**^ (0.048)	−0.250*** (0.056)	0.126^**^ (0.048)	−0.250^***^ (0.056)	0.126^**^ (0.048)	−0.250^***^ (0.056)	0.127^**^ (0.048)	−0.250^***^ (0.056)
**Can avoid COVID-19 infection without being vaccinated**	0.970^***^ (0.042)	1.551^***^ (0.052)	0.968^***^ (0.042)	1.549^***^ (0.052)	0.968^***^ (0.042)	1.547^***^ (0.052)	0.968^***^ (0.042)	1.547^***^ (0.052)
**Country-level variables**								
**Cumulative COVID-19 deaths per 100 million people**								
**Stringency of national measures to suppress COVID-19**								
**GDP growth rate for 2020**								
**Quality of education system**	−0.730 (0.377)	−0.869 (0.511)						
**General trust in government**			−0.018^*^ (0.007)	−0.024^**^ (0.009)				
**Satisfaction with democracy**					−0.015^*^ (0.006)	−0.020^**^ (0.008)		
**Distrust in science**							0.270^*^ (0.124)	0.326^*^ (0.167)
**Proneness to conspiracy theories**								
**Political orientation**								
**Religiosity**								
**Social cohesion**								
**σ^2^**	0.229^***^ (0.066)	0.427^***^ (0.120)	0.207^***^ (0.059)	0.366^***^ (0.104)	0.208^***^ (0.060)	0.373^***^ (0.106)	0.222^***^ (0.064)	0.412^***^ (0.116)
**Variance partition coefficient (VPC)**	0.0651	0.1149	0.0592	0.1001	0.0594	0.1018	0.0632	0.1112
**Covariance**	0.304^***^ (0.086)		0.267^***^ (0.075)		0.270^***^ (0.076)		0.393^***^ (0.083)	
	**Model 9**		**Model 10**		**Model 11**		**Model 12**	
	**Indecisive**	**Refuse vaccination**	**Indecisive**	**Refuse vaccination**	**Indecisive**	**Refuse vaccination**	**Indecisive**	**Refuse vaccination**
**Intercept**	1.536^***^ (0.154)	−1.291^***^ (0.180)	1.532^***^ (0.156)	−1.283^***^ (0.183)	1.540^***^ (0.161)	−1.282^***^ (0.191)	1.540^***^ (0.161)	−1.291^***^ (0.191)
**Gender (RC: Male)**	0.255^***^ (0.038)	0.186^***^ (0.042)	0.255^***^ (0.038)	0.186^***^ (0.042)	0.257^***^ (0.038)	0.186^***^ (0.042)	0.256^***^ (0.038)	0.186^***^ (0.042)
**Age (group centered)**	−0.022^***^ (0.001)	−0.015^***^ (0.001)	−0.022^***^ (0.001)	−0.015^***^ (0.001)	−0.022^***^ (0.001)	−0.015^***^ (0.001)	−0.022^***^ (0.001)	−0.015^***^ (0.001)
**Age when finalizing education (RC:** ** <16)**								
16–19	−0.099 (0.112)	−0.196 (0.118)	−0.098 (0.112)	−0.192 (0.118)	−0.098 (0.112)	−0.197 (0.118)	−0.099 (0.112)	−0.198 (0.118)
20+	−0.301^**^ (0.112)	−0.447^***^ (0.118)	−0.301^**^ (0.112)	−0.443^***^ (0.118)	−0.301^**^ (0.112)	−0.450^***^ (0.118)	−0.305^**^ (0.112)	−0.450^***^ (0.118)
Still studying	−0.415^***^ (0.121)	−0.678^***^ (0.131)	−0.416^***^ (0.122)	−0.675^***^ (0.131)	−0.419^***^ (0.122)	−0.679^***^ (0.131)	−0.419^***^ (0.122)	−0.682^***^ (0.131)
Never had formal education	−0.003 (0.153)	−0.127 (0.169)	−0.005 (0.153)	−0.125 (0.169)	−0.008 (0.153)	−0.126 (0.169)	−0.009 (0.153)	−0.126 (0.169)
**Number of adults in the household (group centered)**	−0.007 (0.015)	0.032^*^ (0.016)	−0.006 (0.015)	0.033^*^ (0.016)	−0.005 (0.015)	0.031 (0.016)	−0.006 (0.015)	0.032^*^ (0.016)
**Place of residence (RC: Rural area)**								
Small or middle–sized town	0.020 (0.048)	−0.254^***^ (0.051)	0.021 (0.048)	−0.253^***^ (0.052)	0.023 (0.048)	−0.255^***^ (0.052)	0.019 (0.048)	−0.253^***^ (0.052)
Large town	−0.166^**^ (0.051)	−0.417^***^ (0.055)	−0.166^**^ (0.051)	−0.415^***^ (0.055)	−0.163^**^ (0.051)	−0.419^***^ (0.055)	−0.170^**^ (0.051)	−0.415^***^ (0.055)
**Living abroad**	0.362^***^ (0.100)	0.182 (0.121)	0.366^***^ (0.101)	0.182 (0.121)	0.360^***^ (0.100)	0.179 (0.121)	0.365^***^ (0.100)	0.177 (0.121)
**Vaccinated in adult age**	−0.508^***^ (0.040)	−0.717^***^ (0.044)	−0.507^***^ (0.040)	−0.717^***^ (0.044)	−0.515^***^ (0.040)	−0.717^***^ (0.044)	−0.511^***^ (0.040)	−0.722^***^ (0.044)
**Seriously ill because of COVID-19**	0.276 ^***^(0.054)	0.224^***^ (0.062)	0.276 ^***^(0.054)	0.225^***^ (0.062)	0.277 ^***^(0.054)	0.226^***^ (0.062)	0.277 ^***^(0.054)	0.224^***^ (0.062)
**Knowing people who were seriously ill because of COVID-19**	−0.412^***^ (0.046)	−0.701^***^ (0.048)	−0.410^***^ (0.045)	−0.700^***^ (0.048)	−0.408^***^ (0.045)	−0.704^***^ (0.048)	−0.411^***^ (0.045)	−0.703^***^ (0.048)
**Satisfaction with the way government has handled the vaccination strategy**	−0.745^***^ (0.040)	−1.499^***^ (0.050)	−0.743^***^ (0.040)	−1.497^***^ (0.050)	−0.744^***^ (0.040)	−1.502^***^ (0.050)	−0.749^***^ (0.040)	−1.502^***^ (0.050)
**Public authorities not sufficiently transparent about COVID-19 vaccines**	0.346^***^ (0.044)	0.412^***^ (0.051)	0.348^***^ (0.044)	0.413^***^ (0.051)	0.352^***^ (0.044)	0.412^***^ (0.051)	0.349^***^ (0.044)	0.415^***^ (0.051)
**Websites provide reliable information on COVID-19 vaccines**	0.029 (0.068)	0.194^**^ (0.072)	0.029 (0.068)	0.194^**^ (0.072)	0.031 (0.068)	0.193^**^ (0.072)	0.031 (0.068)	0.193^**^ (0.072)
**Online social networks provide reliable information on COVID-19 vaccines**	0.052 (0.077)	0.392^***^ (0.078)	0.052 (0.077)	0.392^***^ (0.079)	0.054 (0.077)	0.392^***^ (0.079)	0.052 (0.077)	0.395^***^ (0.079)
**Colleagues, friends and family provide reliable information on COVID-19 vaccines**	0.127^**^ (0.048)	−0.249^***^ (0.056)	0.126^**^ (0.048)	−0.251^***^ (0.056)	0.126^**^ (0.048)	−0.250^***^ (0.056)	0.127^**^ (0.048)	−0.250^***^ (0.056)
**Can avoid COVID-19 infection without being vaccinated**	0.968^***^ (0.042)	1.548^***^ (0.052)	0.967^***^ (0.042)	1.547^***^ (0.052)	0.969^***^ (0.042)	1.550^***^ (0.052)	0.969^***^ (0.042)	1.545^***^ (0.052)
**Country-level variables**								
**Cumulative COVID-19 deaths per 100 million people**								
**Stringency of national measures to suppress COVID-19**								
**GDP growth rate for 2020**								
**Quality of education system**								
**General trust in government**								
**Satisfaction with democracy**								
**Distrust in science**								
**Proneness to conspiracy theories**	0.016^**^ (0.006)	0.021^**^ (0.008)						
**Political orientation**			0.534^*^ (0.222)	0.675^*^ (0.298)				
**Religiosity**					−0.009 (0.007)	−0.008 (0.010)		
**Social cohesion**							0.010 (0.009)	0.010 (0.012)
**σ^2^**	0.197^***^ (0.057)	0.358^***^ (0.102)	0.213^***^ (0.061)	0.390^***^ (0.110)	0.251^***^ (0.072)	0.466^***^ (0.131)	0.256^***^ (0.073)	0.471^***^ (0.132)
**Variance partition coefficient (VPC)**	0.0565	0.0981	0.0608	0.1060	0.0709	0.1241	0.0722	0.1252
**Covariance**	0.257^***^ (0.073)		0.279^***^ (0.079)		0.335^***^ (0.094)		0.338^***^ (0.095)	

* *p < 0.05;*

** *p < 0.01;*

*** *p < 0.001*.

*Estimates based on the multiple imputation technique with 10 imputed values*.

*RC stands for “reference category.”*

*Source: Author's own calculations based on Flash Eurobarometer 494*.

This brings us to Hypothesis 2, which is fully supported by the results of the analysis. That is to say, we found that more educated persons are less likely to have doubts about vaccination (see Table 1). On the other hand, the quality of formal education in a country seems not to directly matter, although the resulting *p*-value is close to the cut-off point for “anti-vaxxers.” An indirect effect, however, must exist given that the prevalence of conspiracy theories in society was also found to significantly influence citizens' views on vaccination. As a matter of fact, trust in information from online sources was identified as the main factor discriminating persons who utterly reject vaccination from the indecisive ones. While hesitant citizens do not find websites and online social networks relevant, these two information channels are vital for the opponents of vaccination.

Another important element distinguishing the two groups is confidence in the information received from friends, colleagues, and relatives. Individuals who tend to trust people they interact with are more inclined to delay vaccination, but at the same time are less likely to fully oppose it. The importance of social ties is further demonstrated by the finding that those who know someone seriously ill from COVID-19 are more positive about vaccination. These results are in favor of Hypothesis 4, at least from a micro-level perspective. The same, however, does not hold on a wider scale given that no significant effect of the variable measuring the level of social cohesion within the country was found.

Further endorsing the role of trust, and offering the most plausible explanation for differences between older democracies and post-socialist societies are the findings related to Hypothesis 3. In short, we found that general trust in government, satisfaction with democratic principles in society, and trust in science, coupled with specific views on the way the authorities have handled the current pandemic are the key determinants of attitudes toward vaccination against COVID-19 in the EU. All coefficients have the expected signs, namely higher trust implies higher readiness to get vaccinated and vice versa.

As already mentioned, there is a large body of literature identifying a weak psychological contract between the state and citizens as the key force behind the rising occurrence of informal practices in post-socialist countries (27, 34). While less harmful forms of noncompliance commonly arise from formal institutions failing to deliver goods and services on time and/or under satisfactory quality, more serious offenses (e.g. undeclared work and akin tax evasion activities) are increasingly the result of rebellion against massive, ineffective, and over-intrusive state apparatus (26–30, 35). Our findings suggest that similar mechanisms are probably in place when it comes to COVID-19 vaccination, meaning that many people in transition societies probably refuse it simply to defy the authorities.

In this respect, one should not neglect the ongoing rise of antiestablishment parties (mostly right-wing ones), which commonly target masses disappointed with the way political leaders sort out current social and economic issues. Indeed, our analysis showed that countries with larger populations of right-oriented citizens face larger resistance to vaccination (Table 1). This endorses the first part of Hypothesis 5. On the other hand, contrary to studies from the rest of the world (21, 22), religious views seem not to represent significant impediments to vaccination in the EU.

**Figure 3:**
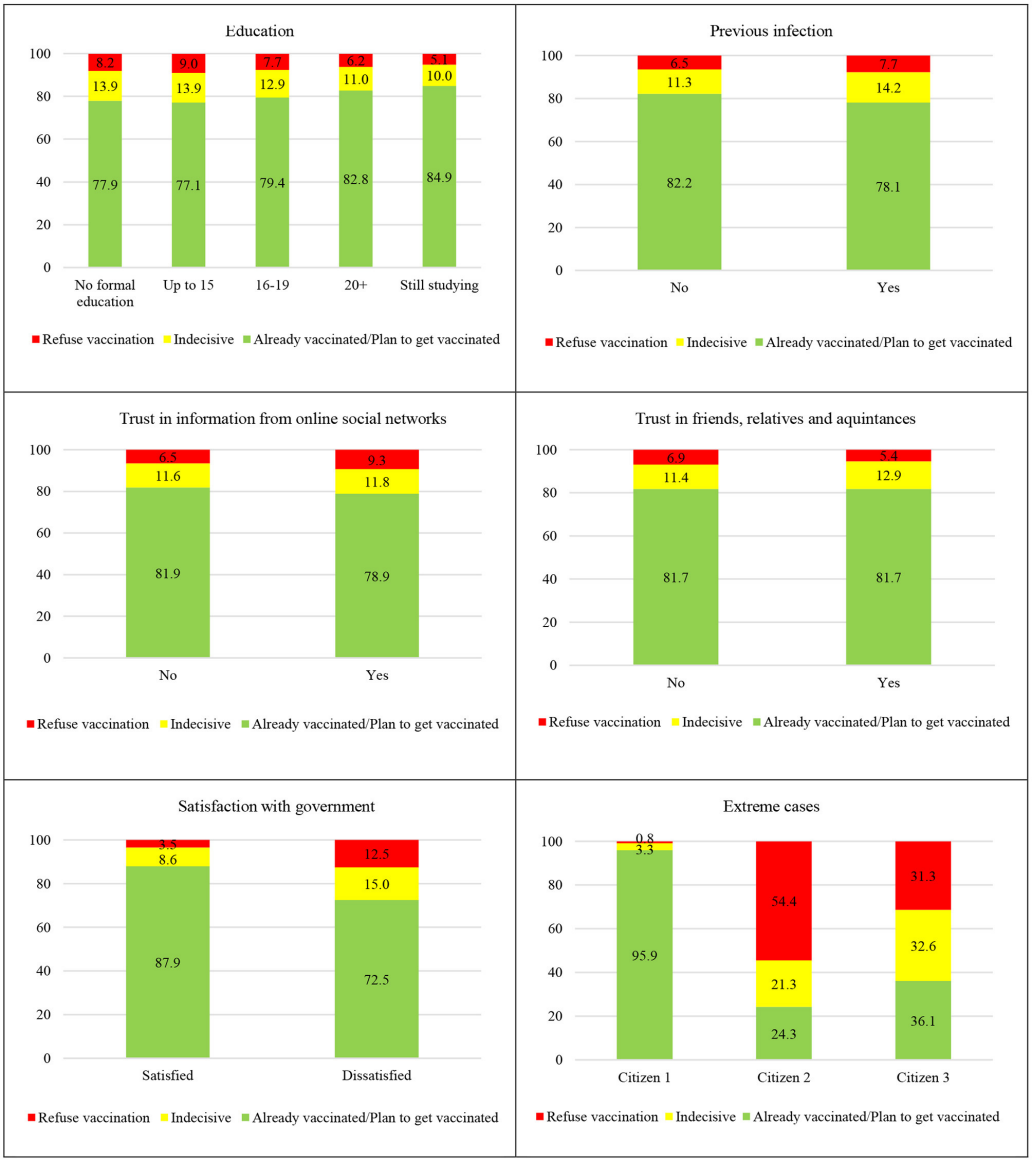
Predicted probabilities by different criteria, %. (1) Panels 1–5 illustrate how the change in the observed variable affects the predicted probabilities for an average EU citizen. (2) Panel 6 shows the predicted probabilities in the extreme scenarios. Citizen 1—was not ill because of COVID-19, but knows people who were seriously ill; satisfied with the way government has handled the vaccination strategy (including the transparency issues); does not rely on friends, relatives, media, or online social networks when seeking information on COVID-19; does not think she/he can avoid infection without vaccination; still studying. Citizen 2 —was ill because of COVID-19, but does not know people who were seriously ill; dissatisfied with the way government has handled the vaccination strategy; relies on media and online social networks when seeking information on COVID-19; thinks she/he can avoid infection without vaccination; finished education by the age of 15. Citizen 3—was ill because of COVID-19, but does not know people who were seriously ill; dissatisfied with the way government has handled the vaccination strategy; relies only on friends and relatives when seeking information on COVID-19; thinks she/he can avoid infection without vaccination; never had formal education. Source: Author's own calculations based on Flash Eurobarometer 494.

To get a more tangible insight into the relevance of individual factors, Figure 3 shows predicted probabilities by five key criteria for each of the three analyzed groups of citizens. The comparison of results straightforwardly highlights the satisfaction with the authorities as by far the most important determinant of vaccination uptake in the EU. As can be seen, individuals dissatisfied with the way the government has handled vaccination strategy are 3.5 times more likely to reject vaccination and 1.7 times more likely to delay it compared to those who are confident about the work of the institutions in charge. The second most important discriminatory factor is trust in information from online social networks, which increases the probability to reject vaccination by almost 50%.

Although individual effects of the remaining covariates are somewhat less salient, each of them represents an important piece of the complex puzzle explaining discrepancies in vaccination rates across the EU. To exemplify this, the last panel of Figure 3 shows the predicted probabilities for three extreme individuals. As illustrated, low-educated persons displeased with the government's strategy against COVID-19, who seek reliable information online and firmly believe they can avoid future infection have a 54.4% chance to be totally against vaccination. This probability reduces to only 0.8% in situations when a person is fully content with the work of the authorities, does not rely on informal sources of information, and has not suffered from severe infection so far (but does know people who did so).

## Discussion

The issue of COVID-19 vaccine hesitancy has so far been approached primarily from the medical perspective. Reflecting knowledge about the factors that led to a reduced interest in vaccination against other diseases, most public discussions and academic studies in the EU and beyond thus paid due attention to a short period within which COVID-19 vaccines were developed and the accompanying lack of reliable safety information (8, 9, 12). However, 1 year into the campaign, it has become clear that safety concerns cannot fully explain strong resistance to COVID-19 vaccination in many EU member states. To explore why this is so, and in particular why the anti-vaccination movement has been more ingrained in post-socialist societies, this paper took a novel approach by scrutinizing the matter through the lens of economic, political, and cultural challenges EU countries are facing at the moment.

For this purpose, data from Flash Eurobarometer 494 on vaccination attitudes in the EU were analyzed. According to the survey, 13.4% of Europeans planned to delay vaccination against COVID-19, while 11.2% did not have any intention to get vaccinated. A deeper insight into the resulting dataset revealed that 15.1% of the variation related to anti-vaccination views and 9.3% of the variation concerning indecisiveness at the EU level go beyond the personal characteristics of survey respondents.

Although the multilevel multinomial logit exposed a range of socio-economic and political factors explaining these variations, the lack of confidence in the state institutions appears to be by far the most important driver of the anti-vaccination movement in many countries. The problem goes beyond the current pandemic and its consequences, given that the conducted analysis highlighted low general trust in government (not necessarily related to the strategies to combat the virus) and dissatisfaction with democracy as key determinants of anti-vaccinationism in the EU. Not only did inconsistencies of the authorities during the pandemic pave the way for the rapid spread of conspiracy theories but they hence most likely further fueled the existing culture of deliberate opposition to formal rules and recommendations (26, 35).

The situation is particularly worrying in post-socialist countries, which are currently witnessing historically low levels of both vertical trust (i.e. trust in the authorities) and horizontal trust (trust in fellow citizens) (36). Previous studies have shown that nepotism, string-pulling, bribery and akin practices inherited from the socialist period still represent a substantial impediment to economic and social development in these “newer democracies” (26, 27, 34). Besides forcing many people to build their own informal networks of trust, weak rule of law has also nurtured the culture of obstruction of the ruling elites. Judging from the results of our analysis, this devastating war is being fought on all possible battlefields, irrespective of the accompanying costs.

The issue of COVID-19 vaccination thus clearly illustrates how the long-lasting structural problems can manifest in unforeseen circumstances if left unaddressed. The combination of defiant behavior and disproportionate reliance on unverified sources of information has undermined the efforts of scientists, healthcare workers, politicians, and compliant citizens to defeat the virus. Consequently, all plans to attain the so-desired herd immunity *via* vaccination have fallen into the water.

However, there are other battles to come, so it is essential to learn the lesson and prevent future escalations of the problem. In line with the findings presented in this paper, as well as from other studies on the misalignment between formal and informal institutions (27, 34, 36), the key emphasis must be on improving the integrity of public institutions. Among other things, this would require increased transparency, improved efficiency of administration, a decisive fight against corruption, and promotion of meritocracy in the public sector. Reforms of education systems also ought to be high on the agenda, whereby the accent should be on the development of critical thinking rather than on a mere task-solving. Finally, endeavors are required to ensure the credibility of information posted online. This primarily applies to online social networks, which are currently the main channel through which conspiracy theories are spread (14, 20, 37).

Although focused on a present-day health issue, this paper is expected to have a wider impact in a variety of scientific fields. Above all, it is envisaged that academics from the fields of political science, economics, psychology, and sociology will find the results presented here interesting and inspiring. Accordingly, if this study encourages other interdisciplinary researchers to further explore this important topic, then it will have fulfilled one of its broader aims.

There are, however, certain limitations of the conducted analysis, which ought to be mentioned. First of all, data utilized in this study were collected during the early phase of vaccination and thus do not necessarily represent the up-to-date state of affairs. The emergence of new variants of the virus on the one hand and perceptible economic consequences of the pandemic on the other (i.e. inflation of consumer prices, shortages of goods and services, new travel restrictions, etc.) have certainly changed the way many people look at the vaccination. In addition, individual-level variables used in the analysis were limited only to those available as part of the Eurobarometer survey, meaning that not all essential covariates were included. Ethnicity, race, marital status, income, and the existence of comorbidities are just the most noteworthy examples of omitted variables. These issues have to be resolved in future studies on the matter, which this paper will hopefully motivate.

## Data Availability Statement

Publicly available datasets were analyzed in this study. This data can be found at: https://search.gesis.org/research_data/ZA7771.

## Author Contributions

The author confirms being the sole contributor of this work and approved it for publication.

## Conflict of Interest

The author declares that the research was conducted in the absence of any commercial or financial relationships that could be construed as a potential conflict of interest.

## Publisher's Note

All claims expressed in this article are solely those of the authors and do not necessarily represent those of their affiliated organizations, or those of the publisher, the editors and the reviewers. Any product that may be evaluated in this article, or claim that may be made by its manufacturer, is not guaranteed or endorsed by the publisher.

## Footnotes

^1^Vaccine hesitancy, which is defined as a delay in acceptance or refusal of vaccination despite availability of vaccination services (1), is not a novel phenomenon. In addition to certain demographic and socio-economic characteristics, the level of one's vaccine hesitancy is also strongly influenced by confidence (in the effectiveness/safety of vaccines, in vaccine providers and health authorities in general), complacency (i.e. perceived risks of vaccine-preventable diseases and/or reasoning about the importance of vaccination), and convenience (availability, affordability and readiness to pay for a vaccine). Since the latter is not relevant for EU citizens, in this paper the focus is only on complacency and confidence.

^2^Notable examples of informal activities that not only stem from, but also contribute to, the state's failure to deliver high-quality goods/services on time are string-pulling, petty corruption in healthcare (i.e. gratuity and gifts to medical practitioners), and undeclared work (26–29). The roots of detachment from the authorities trace back to the period of socialism, which was characterized by vast and inefficient public administration (30–32). The gap additionally deepened during the initial phases of transition due to the flourishing of corruption, clientelism, nepotism and akin practices.

^3^A detailed overview of the methodology can be found in the accompanying report (33).

^4^The resulting dataset contains post-stratification weights, which were employed in our analyses so as to obtain generalized national-level results.

^5^The same procedure was applied in case of explanatory variables with missing values.

^6^More details about explanatory variables are given in Supplementary Table 1.
