# Supplementary material for: What Lies Behind Substantial Differences in COVID-19 Vaccination Rates Between EU Member States?
Source: Front Public Health. 2022 May 26;10:858265. doi: 10.3389/fpubh.2022.858265 (PMC9231480; doi:10.3389/fpubh.2022.858265)
Supplement: Supplementary file 1 [file Table_1.docx]

Supplementary Table 1. An overview of the explanatory variables

|  | **Variable name** | **Type** | **Coding details** | **Remarks** | **Studies finding a significant effect of this or a related covariate** |
| --- | --- | --- | --- | --- | --- |
| **Individual-level variables** | Gender | Binary | 0: Male  1: Female | 86 missing values | (8,10–12,14) |
|  | Age | Interval | Values representing exact age | No missing values | (10,11,13,14,23) |
|  | Age when finalizing education | Categorical | 1: Less than 15  2: 16-19  3: 20+  4: Still studying  5: Never had formal education | 6,125 missing values | (10,14,17–19,37) |
|  | Number of adults in the household | Interval | Values representing exact number of adults in one’s household | 1,751 missing value | (9,20,25,38,39) |
|  | Place of residence | Categorical | 1: Rural area  2: Small or mid-sized town  3: Large town/city | No missing values | (17,21,25,40) |
|  | Living abroad | Binary | 0: Does not live abroad  1: Lives abroad | No missing values | (15,22) |
|  | Vaccinated in adult age | Binary | 0: Received any vaccine in adult age  1: Has not been vaccinated as an adult | 1,574 missing values | (8,9,12,18,41) |
|  | Seriously ill because of COVID-19 | Binary | 0: Person was not seriously ill because of COVID-19  1: Person was seriously ill because of COVID-19 | 1,617 missing values | (14,38,42) |
|  | Knowing people who were seriously ill because of COVID-19 | Binary | 0: Interviewee does not know anybody who was seriously ill because of COVID-19  1: Interviewee knows people who were seriously ill because of COVID-19 | 1,033 missing values | (12,14,40) |
|  | Satisfaction with the way the government has handled the vaccination strategy | Binary | 0: No  1: Yes | 1,491 missing values | (9,18,23,42) |
|  | Public authorities not sufficiently transparent about COVID-19 vaccines | Binary | 0: No  1: Yes | 2,507 missing values | (9,12,13,20,22) |
|  | Websites provide reliable information on COVID-19 vaccines | Binary | 0: No  1: Yes | No missing values | (43,44) |
|  | Online social networks provide reliable information on COVID-19 vaccines | Binary | 0: No  1: Yes | No missing values | (19,43,45) |
|  | Colleagues, friends, and relatives provide reliable information on COVID-19 vaccines | Binary | 0: No  1: Yes | No missing values | (22,39,42) |
|  | Can avoid COVID-19 infection without being vaccinated | Binary | 0: No  1: Yes | 2,439 missing values | (8,10,13,15,37,41) |
| **Country-level variables** | Cumulative COVID-19 deaths per 100 million people | Interval | Figures representing cumulative confirmed COVID-19 deaths per 100 million people | Data refer to 15 May 2021; Source: (45) | (23) |
|  | Stringency of national measures to suppress COVID-19 | Interval | A composite measure based on nine response indicators including school closures, workplace closures, and travel bans, rescaled to a value from 0 to 100 (100 = strictest) | Data refer to 15 May 2021;  Source: (46) | (21,24) |
|  | GDP growth rate for 2020 | Interval | Gross domestic product at market prices, annual growth rates | Source: (47) | (41) |
|  | Quality of education system | Interval | Figures representing average PISA scores for 2018 | Source: (48) | (10,11,14,17,19) |
|  | General trust in government | Interval | The share of the population expressing confidence in the work of government | Figures refer to October 2020; Source: (49) | (10,20,22) |
|  | Satisfaction with democracy | Interval | The share of the population satisfied with the way democracy works in their country | Figures refer to April 2021; Source: (50) | (52,53) |
|  | Distrust in science | Interval | The share of population thinking that we can no longer trust scientists to tell the truth about controversial topics because they depend on money from industry | Figures refer to April-May 2021; Source: (33) | (8,15,20) |
|  | Proneness to conspiracy theories | Interval | The share of population thinking that viruses have been produced in government laboratories to control our freedom | Figures refer to April-May 2021; Source: (33) | (14,20,36) |
|  | Political orientation | Interval | Averaged values of citizens’ political orientations expressed on a scale from 1 (fully left) to 10 (fully right) | Figures refer to September 2019; Source: (53) | (15,43,55) |
|  | Religiosity | Interval | The share of the population attending religious services (apart from weddings, funerals, and christenings) at least once a week | Source: (55) | (21,22,57) |
|  | Social cohesion | Interval | The share of people who feel very attached to their country | Figures refer to February-March 2021; Source: (57) | (9,20) |

Source: Author’s own representation
