# Supplementary figures and images for: What Lies Behind Substantial Differences in COVID-19 Vaccination Rates Between EU Member States?
Source: Front Public Health. 2022 May 26;10:858265. doi: 10.3389/fpubh.2022.858265 (PMC9231480; doi:10.3389/fpubh.2022.858265)

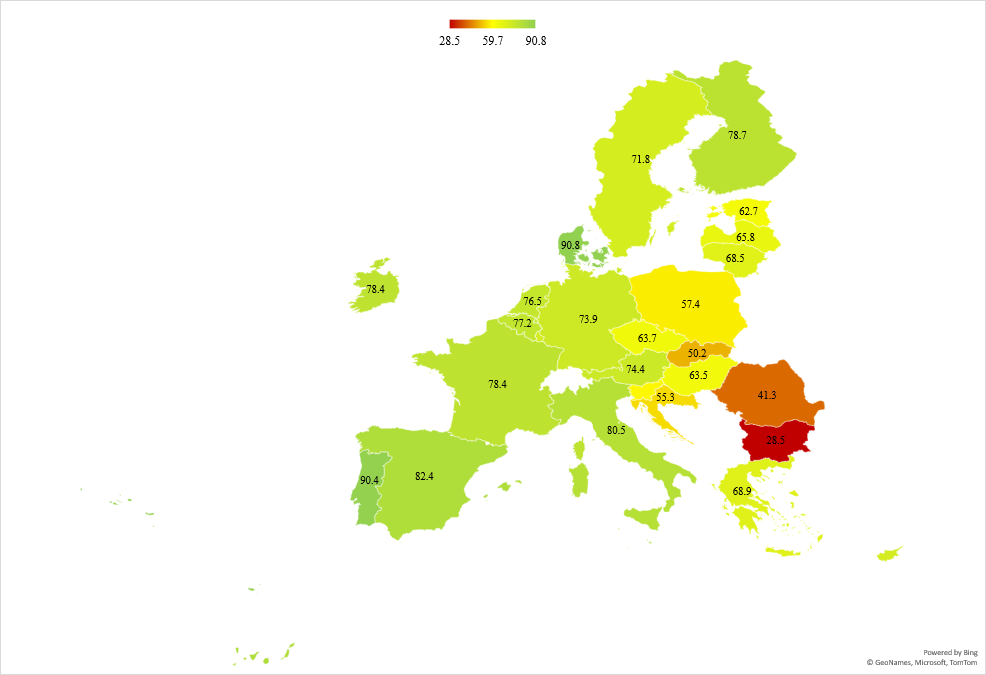


83.4

58.8

72.0

72.2

Supplement: Supplementary file 2 [file Table_2.docx]
